# Thrombocytopenia after meta-iodobenzylguanidine (MIBG) therapy in neuroblastoma patients may be caused by selective MIBG uptake via the serotonin transporter located on megakaryocytes

**DOI:** 10.1186/s13550-021-00823-5

**Published:** 2021-08-23

**Authors:** Thomas Blom, Rutger Meinsma, Franca di Summa, Emile van den Akker, André B. P. van Kuilenburg, Marten Hansen, Godelieve A. M. Tytgat

**Affiliations:** 1 Princess Máxima Center for Pediatric Oncology, Heidelberglaan 25, 3584 CS Utrecht, The Netherlands; 2 Department of Clinical Chemistry, Cancer Center Amsterdam, Amsterdam Gastroenterology Endocrinology Metabolism, Amsterdam University Medical Centers, University of Amsterdam, Meibergdreef 9, 1105 AZ Amsterdam, The Netherlands; 3 Department of Hematopoiesis, Sanquin Research and Landsteiner Laboratory, Amsterdam University Medical Centers, University of Amsterdam, Plesmanlaan 125, 1066 CX Amsterdam, The Netherlands

**Keywords:** Megakaryocytes, Thrombocytopenia, Platelets, Meta-iodobenzylguanidine, MIBG, Neuroblastoma, Serotonin transporter, SERT

## Abstract

**Background:**

The therapeutic use of [^131^I]meta-iodobenzylguanidine ([^131^I]MIBG) is often accompanied by hematological toxicity, primarily consisting of severe and persistent thrombocytopenia. We hypothesize that this is caused by selective uptake of MIBG via the serotonin transporter (SERT) located on platelets and megakaryocytes. In this study, we have investigated whether in vitro cultured human megakaryocytes are capable of selective plasma membrane transport of MIBG and whether pharmacological intervention with selective serotonin reuptake inhibitors (SSRIs) may prevent this radiotoxic MIBG uptake.

**Methods:**

Peripheral blood CD34^+^ cells were differentiated to human megakaryocytic cells using a standardized culture protocol. Prior to [^3^H]serotonin and [^125^I]MIBG uptake experiments, the differentiation status of megakaryocyte cultures was assessed by flow cytometry. Real-time quantitative polymerase chain reaction (RT-qPCR) was used to assess SERT and NET (norepinephrine transporter) mRNA expression. On day 10 of differentiation, [^3^H]serotonin and [^125^I]MIBG uptake assays were conducted. Part of the samples were co-incubated with the SSRI citalopram to assess SERT-specific uptake. HEK293 cells transfected with SERT, NET, and empty vector served as controls.

**Results:**

In vitro cultured human megakaryocytes are capable of selective plasma membrane transport of MIBG. After 10 days of differentiation, megakaryocytic cell culture batches from three different hematopoietic stem and progenitor cell donors showed on average 9.2 ± 2.4 nmol of MIBG uptake per milligram protein per hour after incubation with 10^–7^ M MIBG (range: 6.6 ± 1.0 to 11.2 ± 1.0 nmol/mg/h). Co-incubation with the SSRI citalopram led to a significant reduction (30.1%—41.5%) in MIBG uptake, implying SERT-specific uptake of MIBG. A strong correlation between the number of mature megakaryocytes and SERT-specific MIBG uptake was observed.

**Conclusion:**

Our study demonstrates that human megakaryocytes cultured in vitro are capable of MIBG uptake. Moreover, the SSRI citalopram selectively inhibits MIBG uptake via the serotonin transporter. The concomitant administration of citalopram to neuroblastoma patients during [^131^I]MIBG therapy might be a promising strategy to prevent the onset of thrombocytopenia.

**Supplementary Information:**

The online version contains supplementary material available at 10.1186/s13550-021-00823-5.

## Background

Neuroblastoma, the most common extracranial solid tumor of childhood, originates from the early developing embryonic sympathetic nervous system [1]. Approximately 90% of neuroblastomas express the norepinephrine transporter (NET) [2], enabling the application of meta-iodobenzylguanidine (MIBG), a structural analogue of norepinephrine, for imaging and treatment purposes. MIBG radiolabeled with iodine-123 is used as a highly NET-selective imaging radiopharmaceutical [3], whereas MIBG radiolabeled with iodine-131 is being used since 1984 as a therapeutic radiopharmaceutical in patients with neuroblastoma [4].

Over the years, different strategies have been studied to optimize the application of [^131^I]MIBG therapy in neuroblastoma patients [5]. In the majority of these studies, [^131^I]MIBG was used as salvage therapy for refractory or relapsed disease [6]. However, in the Netherlands [^131^I]MIBG has also been extensively used upfront for induction therapy [7, 8]. Pain relief in the palliative setting is another application of [^131^I]MIBG [9]. Despite the fact that [^131^I]MIBG therapy has been used for 37 years in the treatment of neuroblastoma patients, the definitive answer on the optimal role and timing of [^131^I]MIBG therapy is not yet given and awaits further prospective trials. Currently, the Children's Oncology Group (COG) is recruiting newly diagnosed high-risk neuroblastoma patients for a randomized Phase 3 study, in which patients receive either standard induction or induction with the addition of [^131^I]MIBG therapy (NCT03126916) [10]. At the same time in Europe, SIOPEN (International Society of Pediatric Oncology, European Neuroblastoma) is investigating whether [^131^I]MIBG therapy has a role in intensification treatment strategies for high-risk patients with a poor response to induction chemotherapy (VERITAS study; NCT03165292). A complete novel application is the combined use of [^131^I]MIBG therapy with targeted, antibody-based immunotherapy in relapsed and refractory patients. Examples are safety and efficacy studies of [^131^I]MIBG therapy with dinutuximab (anti-GD2 antibody; NCT03332667) and with the combination of dinutuximab and Nivolumab (anti-Programmed Cell Death Protein 1; MiNivAN study, NCT02914405).

An unwanted adverse effect of the therapeutical application of [^131^I]MIBG has been hematological toxicity, mainly consisting of severe and persistent thrombocytopenia [11–14]. Thrombocytopenia in the pediatric oncology population may lead to interruption of therapy, platelet transfusions, and bleeding complications [15]. It is conceivable that radiation exposure after selective uptake of MIBG by megakaryocytes is the major cause of MIBG therapy-associated thrombocytopenia. Both human megakaryocytes [16, 17] and their offspring cells, platelets [18], express the serotonin transporter (SERT) on their cell membrane. SERT-mediated MIBG uptake by human platelets has previously been described [19]. More recently, we have demonstrated that selective serotonin reuptake inhibitors (SSRIs) are able to prevent radiotoxic MIBG uptake in platelets without affecting uptake in neuroblastoma tumor [20]. However, two studies conducted in 1995 and 2002, respectively, have failed to demonstrate MIBG uptake in vitro in megakaryocytic cell lines [21] and in cultured human megakaryocytes [22]. Over the years, vast progression has been made in the in vitro culture and differentiation of human megakaryocytes [23, 24]. The aim of our current study was to investigate whether cultured human megakaryocytes, using an optimized method of differentiation, are capable of selective MIBG uptake.

## Methods

### Megakaryocytic cell culture protocol/differentiation

Megakaryocytic cells were cultured in suspension using a standardized differentiation protocol, as previously described [23, 25, 26]. In short, CD34^+^ hematopoietic stem and progenitor cells (HSPCs) were isolated from mobilized peripheral blood of healthy adult donors. Informed consent was given in accordance with the Declaration of Helsinki and the Dutch national and Sanquin internal ethical review boards. CD34^+^ cells were differentiated to megakaryocytes in modified Iscove's Modified Dulbecco's Media (Cellquin) [27] supplemented with 50 ng/ml stem cell factor (SCF, produced at Sanquin, Amsterdam, the Netherlands), 50 ng/ml Nplate (agonist of the thrombopoietin receptor (MPL), Amgen, Thousand Oaks, USA), 100 ng/ml FLT-3 (#78,009, Stemcell Technologies, Vancouver, Canada) and 20 ng/ml IL-6 (#78,050, Stemcell Technologies) for 4 days, followed by culturing in 50 ng/ml Nplate and 10 ng/ml IL-1β (#200-01B, PeproTech, Cranbury, USA) for 5 additional days.

On day 9, prior to [^3^H]serotonin and [^125^I]MIBG uptake experiments, differentiation status of megakaryocyte cultures was assessed by flow cytometry (LSR-II, BD Biosciences, San Jose, USA) with antibodies against CD34 APC (1:400, #343510, BioLegend, San Diego, USA), CD41a (1:800, #303718, Biolegend), CD42b FITC (1:50, #853.231.010, Diaclone, Besançon France), and CD42a PE (1:200, #558819, BD Biosciences) [28].

### SERT and NET mRNA expression using real-time quantitative polymerase chain reaction (RT-qPCR)

RNA was isolated from megakaryocytic cell cultures with TRIzol reagent (ThermoFisher, Carlsbad, USA) according to the manufacturer’s instructions on 8 different time points (day 0 – 14 of culture). Methods for cDNA synthesis and RT-qPCR reaction performed on the StepOnePlus (Applied Biosystems) were described previously [29]. To control for DNA input and PCR-efficiency, NET and SERT expression was normalized to β-glucuronidase (GUS) expression using the following equation: normalized cycle threshold (ΔC_T_) = (C_T_ GUS − C_T_ NET/SERT) [30]. A sample was scored negative if the C_T_ NET/SERT value was 40 or greater. All qPCR experiments were carried out in duplicate for GUS and in triplicate for NET/SERT, and mean values were used for analysis. ΔC_T_ values are expressed as mean ± SD.

Primers and probes for NET and SERT were designed using Primer Express version 1.5 software (Applied Biosystems, Foster City, CA) and Oligo 6 (Molecular Biology Insights, Cascade, USA), based on published gene sequences (ENSEMBL: NET transcript ENST00000568943.5; SERT transcript ENST00000650711.1). The amplicons spanned introns of more than 500 bp, and no amplification of genomic DNA was observed. Oligonucleotides were obtained from Eurogentec (Liège, Belgium) (see Table 1 for sequences). The primer/probe combination for glucuronidase-β (GUS) has been published previously [31].

**Table 1:** Primer and probe combinations

NET (*SLC6A2*)	Norepinephrine transporter	Exon
Forward primer Reverse primer Probe	5′-TGG-CTC-CGT-GCT-TGG-C-3′ 5′-CCG-CTG-CTC-TCG-TGA-AGG-3′ 5′FAM-CCG-AGT-TTT-ATG-AGC-GTG-GTG-TCC-TGC-3′TAMRA	4 5 4–5
SERT (*SLC6A4*)	Serotonin transporter	
Forward primer Reverse primer Probe	5′-AAG GAA ATG CTC GGC TTC AG-3′ 5′-GGG CTC ATC AGA AAA CTG CAA-3′ 5′FAM-CCA TCA GCC CTC TGT TTC TCC TGT TCA TC-3′TAMRA	13 14 13–14

### [^3^H]serotonin and [^125^I]MIBG uptake experiments

All cells from the megakaryocytic cultures were collected by slowly resuspending the cultures with a 10 ml pipet on day 9 of culture. Cells were counted and brought to a concentration of 1.5 × 10^6^ cells per 2 ml, before seeding in 6 wells (9.5 cm^2^ each) plates in fresh differentiation medium. After 24 h (day 10 of culture), fresh medium containing MIBG or serotonin with a final concentration of 10^–7^ M and a fixed concentration of radioactivity of 3.7 kBq/ml was added. Part of the samples were co-incubated with the SSRI citalopram at a final concentration of 1 µM. After one hour incubation for serotonin and four hours for MIBG at 37 °C and 5% CO_2_, substrate availability in the incubation medium was > 98% for MIBG and > 86% for serotonin under these experimental conditions. The well content was transferred to 15 ml centrifuge tubes. Wells were washed twice with 1 ml Hank’s Balanced Salt Solution (HBSS), and this washing fluid was added to the tubes. The cells were collected by centrifugation for 5 min at 150×g at room temperature (Hettich Rotanta 460, Tuttlingen, Germany). Cell pellets were washed twice with 1 ml of HBSS and centrifuged. For samples incubated with [^3^H]serotonin, cell-associated radioactivity was extracted with 0.2 M sodium hydroxide and counted in Ultima Gold® (PerkinElmer, Waltham, USA) scintillation fluid with a PerkinElmer Tri-Carb liquid scintillation counter (4910TR). Samples incubated with [^125^I]MIBG were counted using a gamma counter (PerkinElmer WIZARD^2^ automatic γ-counter). Total uptake was expressed as nanomole of substrate uptake per milligram protein per hour.

### Uptake studies of [^3^H]serotonin and [^125^I]MIBG in transfected HEK293 cells

NET (addgene plasmid #15475) and SERT (addgene plasmid #15483) were transiently expressed in HEK293 cells (ATCC® CRL-3216™, Manassas, USA) as previously described [20]. Two days after plating the transfected HEK293 cells in 6 wells plates, at a confluence of 80 – 90%, the culture medium was removed and fresh medium containing MIBG or serotonin with a final concentration of 10^–7^ M and a fixed concentration of radioactivity of 3.7 kBq/ml was added. Part of the samples were co-incubated with the SSRI citalopram [1 µM]. Nonspecific uptake was determined in HEK-293 cells transfected with an empty vector alone. After one hour of incubation at 37 °C, the cells were washed twice with HBSS, after which the cell-associated radioactivity was counted as described above.

### (Radio) chemicals

The SSRI citalopram (citalopram-HBr) was obtained from Sigma-Aldrich (St. Louis, USA). [^125^I]MIBG (specific activity ≈ 0.65 TBq/mmol) was purchased from Chelatec (Saint-Herblain, France). [^3^H]Serotonin (5-[^3^H]Hydroxytryptamine Creatinine Sulfate, specific activity ≈ 3.84 TBq/mmol) was purchased from PerkinElmer.

*Data analysis.* Unless stated otherwise, results are expressed as mean ± SD (n). Results were statistically analyzed using the Student's t-test and were considered significant if p < 0.05.

## Results

### Expression of SERT and NET in cultured human megakaryocytes

**Fig.1:**
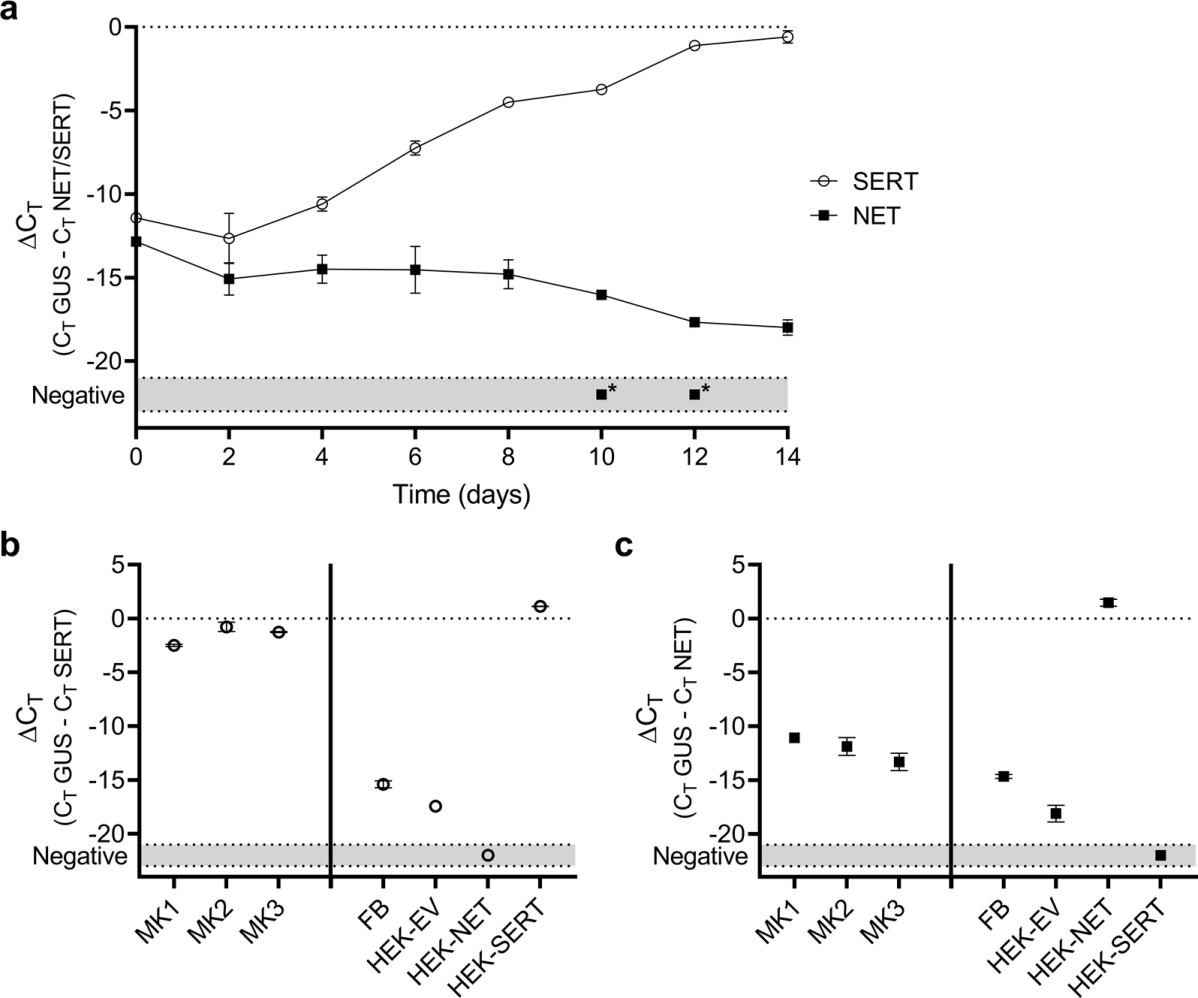
mRNA expression of SERT and NET in megakaryocytic cells evaluated using RT-qPCR. **a** mRNA expression of SERT and NET in CD34^+^ HSPCs undergoing megakaryocytic differentiation. Cells were cultured for 14 days, SERT and NET mRNA expression was evaluated every two days. **b** SERT mRNA expression of megakaryocytic cell culture batches from three different HSPC donors (MK1-3) after 10 days of differentiation. HEK-EV, HEK-NET, HEK-SERT are HEK293 cells were transfected with an empty vector, NET, or SERT, respectively, and served, in combination with fibroblasts (FB) as controls. **c** NET mRNA expression of MK1-3 after 10 days of differentiation. NET mRNA expression was also determined in fibroblasts (FB) and HEK293-EV, HEK293-NET, and HEK293-SERT. Results are expressed as mean ± SD (n = 2, experiments in triplicate). *If a ΔC_T_ could not be calculated due to C_T_ values of SERT or NET > 40 cycles, the sample was scored ‘Negative’

To investigate the mRNA expression of SERT and NET in CD34^+^ hematopoietic stem and progenitor cells (HSPCs) undergoing megakaryocytic differentiation, CD34^+^ HSPCs from two healthy adult donors were cultured for 0–14 days. SERT and NET mRNA expression were evaluated using RT-qPCR every two days. The marker expression was normalized to the housekeeping gene GUS, which expression was stable for all time points with a median of 22.0 (range 19.5–23.8). On day zero prior to megakaryocytic differentiation, both SERT and NET expression in HSPCs are low with a mean ΔC_T_ value of -12.1 (± 1.0) (Fig. 1a). In the subsequent days of megakaryocytic differentiation, NET expression reduces even further to a ΔC_T_ value of − 18.0 (± 0.5) on day 14. On the other hand, SERT expression increased in time and reached a ΔC_T_ value − 0.6 (± 0.4) on day 14, matching the expression of housekeeping gene GUS at the final stages of megakaryocytic differentiation.

On day 0, 20 × 10^6^ CD34^+^ HSPCs from three different healthy adult HSPC donors (MK1-3) were taken into culture and used for further experiments. The next experiments were performed using these megakaryocytic cell culture batches after 10 days of differentiation. Prior to the start of [^3^H]serotonin and [^125^I]MIBG uptake experiments, mRNA expression of SERT and NET was determined. Again, SERT is highly expressed in all three batches (MK1-3) with a mean ΔC_T_ value of − 1.5 (± 0.9) (Fig. 1b) and is comparable to SERT expression in the SERT-transfected HEK293 cells (ΔC_T_ = 1.1 (± 0.2)). The highest SERT mRNA expression was observed in batch MK2 (ΔC_T_ = − 0.8 (± 0.4)), while the lowest SERT expression was observed in batch MK1 ((ΔC_T_ = − 2.5 (± 0.1)). As expected, SERT mRNA expression was either low or undetectable in NET-transfected HEK293 cells, and HEK293 cells were transfected with an empty vector, and fibroblasts, which served as a non-neural crest-derived control cell line.

NET mRNA expression was also evaluated using RT-qPCR (Fig. 1c). Overall, NET expression was low in all three megakaryocytic cell culture batches with a mean ΔC_T_ value of -12.1 (± 1.1). High NET expression was only observed in NET-transfected HEK293 cells (ΔC_T_ = 1.4 (± 0.3). Low or undetectable expression of NET was observed in SERT-transfected HEK293 cells, HEK293 cells transfected with an empty vector, and fibroblasts.

### Evaluation of the differentiation status of cultured human megakaryocytes using flow cytometry

**Fig.2:**
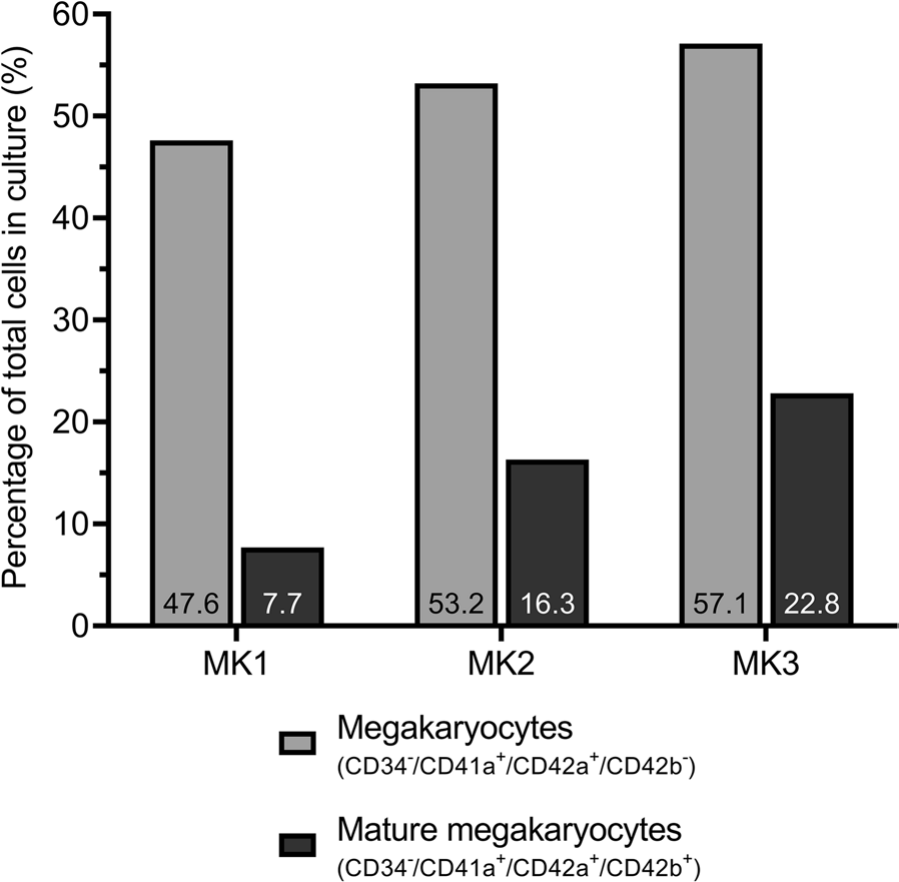
The differentiation status of megakaryocytes in three cell culture batches (MK1-3) assessed by flow cytometry. Of the three megakaryocytic cell culture batches from three different healthy adult HSPC donors (MK1-3), MK3 has both the most megakaryocytes and the most mature megakaryocytes after 9 days of differentiation. The results were based on flow cytometry expression of the cell surface antigens CD34, CD41a, CD42a, a marker of true megakaryocytic commitment, and CD42b, a marker of more mature megakaryocytes

On day 9 of differentiation, 24 h before [^3^H]serotonin and [^125^I]MIBG uptake experiments, differentiation status of the three megakaryocytic cell culture batches (MK1-3) was assessed by flow cytometry. In addition to CD34 expression, we determined expression of CD41a and CD42a, the latter is also known as glycoprotein IX (GP9), as markers of true megakaryocytic commitment [32]. CD42b, also known as glycoprotein Ib, was used for the identification of more mature megakaryocytes. Flow cytometric analysis of the three batches confirmed that our standardized differentiation protocol led to proper maturation of megakaryocytes (Fig. 2 and Additional file 1: Figure S1a for flow cytometry plots), with 55.3% (47.6% + 7.7%, MK1) to 79.9% (= 57.1% + 22.8%, MK3) of cells with a megakaryocytic phenotype. Of the three megakaryocytic cell culture batches, MK3 demonstrated the strongest development toward maturation with the most megakaryocytes (57.1%; CD34^−^/CD41a^+^/CD42a^+^/CD42b^−^) and most mature megakaryocytes (22.8%; CD34^−^/CD41a^+^/CD42a^+^/CD42b^+^). Only 47.6% and 7.7% of cells reached those stages of maturation after 9 days of cell culture differentiation in MK1. In Additional file 1: Figure S1b + c, a normal CD antigen expression pattern in time during megakaryocytic differentiation is shown.

### *Uptake and inhibition of [*^*3*^*H]serotonin and [*^*125*^*I]MIBG in cultured human megakaryocytes*

**Fig.3:**
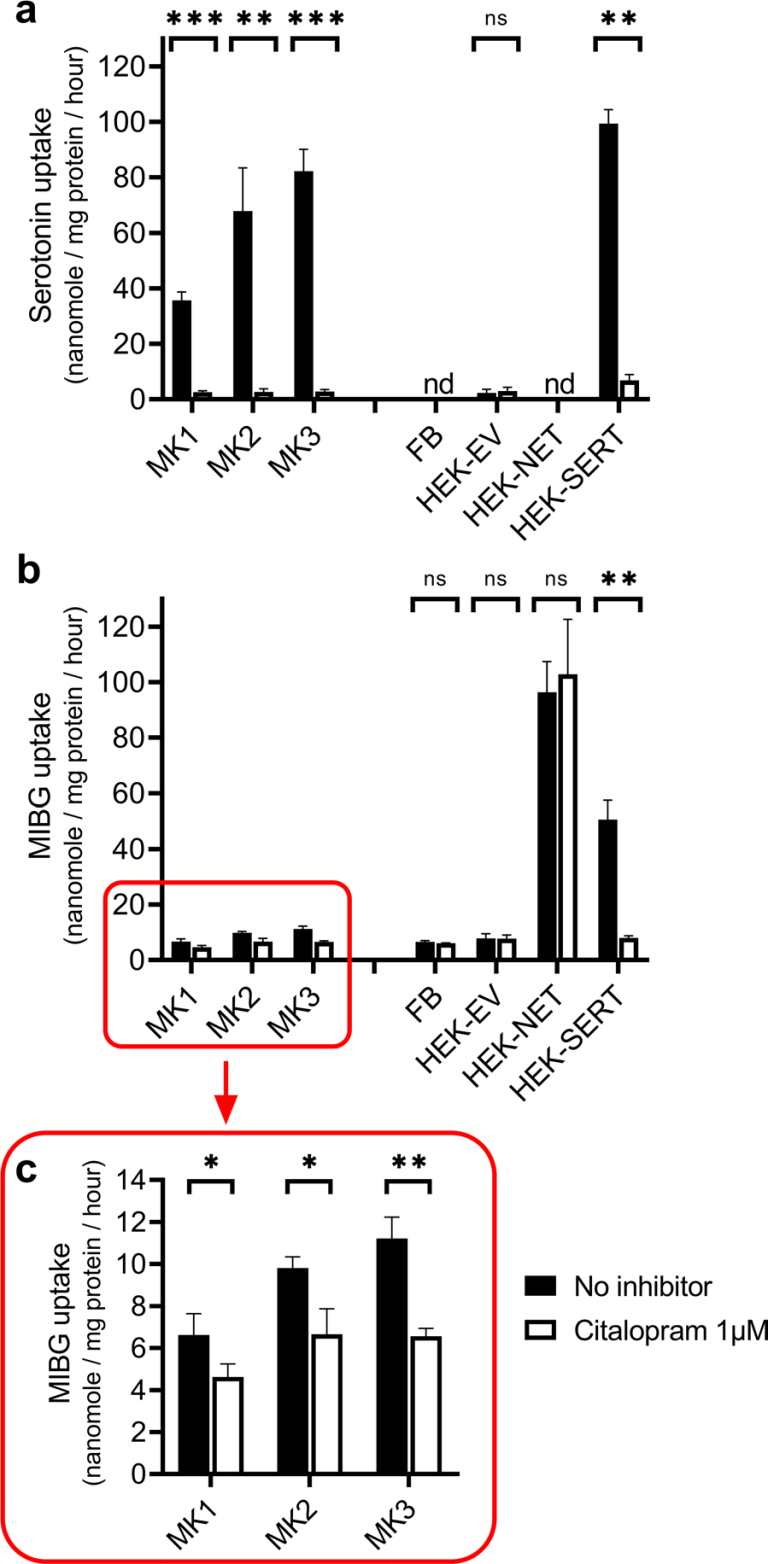
Uptake of serotonin and MIBG in cultured human megakaryocytes (MK1-3) after 10 days of differentiation and in SERT- and NET-transfected HEK293 cells. Samples were co-incubated with no inhibitor (black bars) or with the SSRI citalopram (1 μM; white bars). **a** Serotonin uptake in three megakaryocytic cell culture batches from three different healthy adult HSPC donors (MK1-3), fibroblasts (FB), and HEK293 cells transfected with an empty vector (HEK-EV), NET (HEK-NET), or SERT (HEK-SERT). **b** MIBG uptake in MK1-3, fibroblasts (FB), transfected HEK293 cells (HEK-EV, HEK-NET, HEK-SERT). **c** Close-up view of panel B showing MIBG uptake in MK1-3. The difference in MIBG uptake between samples with and without citalopram was significant in all three batches (MK1: p = 0.043, MK2: p = 0.014, MK3: p = 0.002). Data represent mean + SD. Please note the different scales of the Y-axis. Asterisks were used to indicate significance levels: p < 0.05 (*), p ≤ 0.01 (**), and p ≤ 0.001 (***). Serotonin/MIBG uptake is expressed as nanomole of substrate uptake per milligram protein per hour

Uptake experiments with distinct cell populations, sorted with fluorescence-activated cell sorting (FACS) after 10 days of differentiation, according to expression of CD34, CD41a, CD42a, and CD42b, were not feasible due to biological fragility induced by mechanical stress. Therefore, experiments were performed with all cells from the megakaryocytic cultures after 10 days of differentiation. To investigate whether megakaryocytes have a functional serotonin transporter (SERT), uptake experiments were performed using the natural ligand serotonin. In these experiments, cultured human megakaryocytes from all three badges (MK1-3) showed the capacity for uptake of [^3^H]serotonin. The total uptake (nanomole of serotonin uptake per milligram protein per hour) ranged from 35.7 ± 3.0 nmol/mg/h for MK1 to 82.2 ± 7.9 nmol/mg/h for MK3 after incubation with 10^–7^ M serotonin (Fig. 3a). Moreover, citalopram, a selective inhibitor of the serotonin transporter, almost completely reduced this serotonin uptake to 2.7 ± 0.8 nmol/mg/h, demonstrating that serotonin uptake was SERT specific and > 92% citalopram sensitive. The total serotonin uptake in SERT- transfected HEK293 cells is 99.3 ± 5.0 nmol/mg/h, and addition of citalopram led to a reduction of 93% in uptake to 6.8 ± 2.1 nmol/mg/h. HEK293 cells transfected with an empty vector showed minimal uptake of serotonin (2.4 ± 1.2 nmol/mg/h); moreover, coincubation with citalopram had no effect (3.0 ± 1.3 nmol/mg/h).

Overall, [^125^I]MIBG uptake in cultured human megakaryocytes (MK1-3) was lower in comparison with the natural ligand serotonin. The total uptake (nanomole of MIBG uptake per milligram protein per hour) ranged from 6.6 ± 1.0 nmol/mg/h for MK1 to 11.2 ± 1.0 nmol/mg/h for MK3 after incubation with 10^–7^ M MIBG (Fig. 3b). In the samples co-incubated with citalopram, a reduction in MIBG uptake was seen ranging from 30.1% (MK1) to 41.5% (MK3). The difference in uptake between samples with and without citalopram was significant in all three batches (Fig. 3c), demonstrating SERT-specific uptake of MIBG. The highest MIBG uptake was observed in NET-transfected HEK293 cells (96.3 ± 11.1 nmol/mg/h), which served here as positive control. As expected, the SSRI citalopram had no effect on NET-mediated MIBG uptake in NET-transfected HEK293 cells (102.8 ± 19.8 nmol/mg/h). MIBG uptake in SERT-transfected HEK293 cells was 50.6 ± 6.9 nmol/mg/h. Co-incubation with citalopram of SERT-transfected HEK293 cells led to a significant reduction in MIBG uptake (8.0 ± 0.8 nmol/mg/h; p = 0.003). MIBG uptake in fibroblasts and HEK293 cells transfected with an empty vector was low and not SERT-mediated, given that addition of citalopram did not lead to a reduction in MIBG uptake.

**Fig.4:**
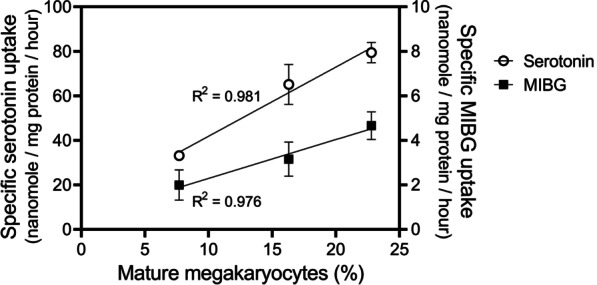
Relation between mature megakaryocytes and specific substrate uptake. The specific substrate uptake of serotonin (left y-axis) and MIBG (right y-axis) was plotted against the percentage of mature megakaryocytes (CD34^−^/CD41a^+^/CD42a^+^/CD42b^+^) in the culture batch. Data from megakaryocytic cell culture batches from three different healthy adult HSPC donors (MK1-3) are plotted (mean ± SD)

As the percentage mature megakaryocytes varied considerably between the three batches of cultured human megakaryocytes (between 7.7 and 22.8%, respectively), we investigated whether a correlation existed between the percentage mature megakaryocytes and MIBG and serotonin uptake. For all three batches (MK1-3), we calculated the specific serotonin and MIBG uptake, that is the total uptake minus uptake with citalopram inhibition, the latter representing non-SERT-selective accumulation. Specific uptake for both serotonin and MIBG was highest in MK3 and lowest in MK1. When the specific serotonin and MIBG uptake were plotted against the percentage of mature megakaryocytes in the culture batch (Fig. 4), a strong positive correlation was observed. To conclude, we demonstrated SERT-mediated uptake of both serotonin and MIBG in cultured megakaryocytes. Our data suggest that more mature megakaryocytes are better equipped to accumulate serotonin and MIBG than megakaryocytes at earlier stages of differentiation.

## Discussion

The most common side effect encountered after [^131^I]MIBG therapy is hematological toxicity, in particular thrombocytopenia [33]. Since platelets are anucleate cells with a finite lifespan of 9–11 days [34], and the observed thrombocytopenia is a delayed toxicity which can persevere for months [11], we investigated whether platelet precursor cells, i.e., megakaryocytes located in the bone marrow, are the primary targets of radiotoxic MIBG. To date, only circumstantial evidence exists for the capacity of megakaryocytic cells to concentrate MIBG in the form of a case report demonstrating osteomedullary MIBG uptake in a 13-month-old patient with acute megakaryocytic leukemia [35]. Since both the norepinephrine transporter (NET) and serotonin transporter (SERT) have the capacity for MIBG uptake [19, 20], we investigated whether SERT, located on the cell membrane of human megakaryocytes [16, 17], is capable of MIBG uptake in megakaryocytes and whether citalopram, a specific inhibitor of the serotonin transporter (SSRI), could inhibit this uptake.

Our study demonstrated that in vitro suspension-cultured human megakaryocytes using a standardized differentiation protocol are capable of selective plasma membrane transport of MIBG. After 10 days of differentiation, megakaryocytic cell cultures from three different healthy adult HSPC donors showed on average 9.2 ± 2.4 nmol of MIBG uptake per milligram protein per hour after incubation with 10^–7^ M MIBG (range: 6.6 ± 1.0 nmol/mg/h to 11.2 ± 1.0 nmol/mg/h). In comparison, serotonin uptake was on average 61.99 ± 23.8 nmol per milligram protein per hour after incubation with 10^–7^ M serotonin (range: 35.7 ± 3.0 nmol/mg/h to 82.2 ± 7.9 nmol/mg/h). Co-incubation with the SSRI citalopram led to a significant reduction (30.1–41.5%) in MIBG uptake, implying SERT-specific uptake of MIBG. A strong correlation between the number of mature megakaryocytes, expressing glycoprotein Ib (CD42b) [36] assessed by flow cytometry phenotyping, and specific substrate uptake (both MIBG and serotonin) was discovered. This suggests that the most mature megakaryocytes have the highest uptake capacity for MIBG. Unfortunately, it was not possible to perform uptake and inhibition experiments with purified mature megakaryocytes, as the fragility of these cells prevented sorting via, e.g., FACS or MACS (magnetic-activated cell sorting).

Our results demonstrated for the first time the selective uptake of MIBG via the serotonin transporter by cultured human megakaryocytes and thereby offer an alternative explanation to the hematologic toxicity encountered after ^131^I-MIBG therapy. Early studies reported that the whole-body radiation dose could serve as an excellent predictor of thrombocytopenia after therapeutic ^131^I-MIBG [37, 38], and this association was confirmed by more recent studies [11, 39, 40]. Although a large study performed by Trieu et al. could not find significant association between both entities, this was attributed to the high-level and narrow range of MIBG activity applied (16.3 mCI/kg ± 5.9 [mean ± SD]). However, the whole-body radiation dose cannot explain the thrombocytopenia encountered in neuroblastoma patients treated with ^125^I-MIBG, a radiopharmaceutical emitting Auger electrons with an insufficient range to interact with surrounding untargeted cells [41], which was more severe than in ^131^I-MIBG-treated patients in the same study who received higher radiation doses [38]. Our finding that megakaryocytes are capable of selective MIBG uptake may explain these findings. Our group described earlier that peripheral blood stem cell (PBSC) apheresis is feasible after upfront ^131^I-MIBG therapy in neuroblastoma patients, although delayed platelet reconstitution occurred after reinfusion in MIBG-treated patients in comparison with chemotherapy-only-treated patients [42]. Interestingly, no association was found between bone marrow tumor infiltration at diagnosis and platelet reconstitution. We hypothesize that ^131^I-MIBG taken up by megakaryocytes in the bone marrow might damage neighboring hematopoietic stem cells via cross-fire irradiation and radiation-induced biological bystander effects [41, 43], leading to delayed platelet reconstitution after reinfusion. These two radiobiological effects may also partly account for the widespread and enduring hematological toxicity seen after ^131^I-MIBG therapy.

Our results are in apparent contrast with a previous study by Tytgat et al. showing the inability of specific uptake of MIBG in cultured megakaryocytes [22]. It is conceivable that the discrepancy between our results and those reported by Tytgat et al. is due to differences in differentiation protocols which may have led to less mature megakaryocytes and therefore less SERT expression on the cell surface. First, the composition of the differentiation medium used is different. It is known that culture medium composition is critical for proper differentiation and maturation of HPSCs [27]. Second, while Tytgat et al. used thrombopoietin as sole growth factor, without the addition of cytokines [22], our optimized and reproducible differentiation protocol [23, 25] includes, besides Nplate, a thrombopoietin analog, stem cell factor (SCF), and the combination of cytokines interleukin-1β and -6 (IL-1β and IL-6). Third, the differentiation of the megakaryocyte cultures was determined solely based on the expression of CD61, or glycoprotein IIIa, a non-specific surface marker present on platelets, megakaryocytes, monocytes, macrophages, and endothelial cells [44]. While CD61 expression is associated with commitment to the megakaryocytic lineage, it is not a specific marker for megakaryocytic maturation [45]. It is, therefore, unclear whether and to what extent the differentiation protocol led to proper maturation of megakaryocytes in this study [22].

The MIBG uptake in megakaryocytic cultures proved to be 5.4–7.3-fold lower compared with the serotonin uptake. Previously, we showed that the affinity of SERT for MIBG was considerable lower than for the natural ligand serotonin (K_M_ 9.7 µM and K_M_ 3.6 µM, respectively) [20]. Moreover, the catalytic efficiency of SERT, defined as the ratio $\frac{{V}_{max}}{{K}_{M}}$, was 4.2 × higher for serotonin than for MIBG, implying that SERT has a substantial higher ability to transport serotonin than MIBG. Interactions between SERT protein and ligand may account for these observed differences. Recently, critical interactions between SERT protein and ligand were elucidated [46–48]. These interactions provide a structural explanation for the observed uptake differences of serotonin and MIBG by SERT. Although, on the one hand, striking similarities in interactions between the transporter protein and ligands are evident among the neurotransmitter:sodium symporter (NSS) family that includes both SERT and NET [47–49]. There are other SERT protein–ligand interactions, on the other hand, that are critical for membrane transport [47], that can only be established by a bicyclic indole compound as serotonin and not by the catecholamine-related MIBG.

Another mechanism that may lead to the observed differences in serotonin and MIBG uptake in megakaryocytes is that plasma membrane SERT density and, subsequently, serotonin uptake, increases after exposure to increasing extracellular serotonin levels. This regulatory mechanism was first discovered in platelets [50, 51]. Whether a similar regulatory mechanism exists in megakaryocytes, and whether exposure to MIBG leads to similar up-regulatory effects on SERT, is not known.

Differences in SERT transporter expression might explain why the MIBG uptake in megakaryocytic cultures was 4.5–7.6-fold lower than the MIBG uptake in HEK293-SERT transfected cells. Caution should be exercised, however, when comparing a highly heterogeneous population of cells, i.e., the megakaryocytic cell cultures derived from healthy adult HSPC donors after 10 days of differentiation, with a homogenous population of transfected HEK-cells overexpressing SERT. This difference between heterogeneous and homogenous cell populations might also explain why the heterogeneous, megakaryocyte cultures in our uptake experiments were not “able to concentrate large amounts of administered MIBG (…) equivalent to that observed for serotonin” as homogenous, donor-derived platelets were in earlier studies [19]. Furthermore, the difference in uptake capacity between platelets and their progenitor megakaryocytes might be explained by the absence of dense granules (DG) in the latter cells. DGs have the capacity to accumulate monoamines, like serotonin [50], but also catecholamine-related substances as MIBG [52]. For a long time, DGs were thought to be fully matured within megakaryocytes [53, 54]. Recent evidence suggested that DGs are not formed in megakaryocytes but instead in proplatelets [55]. Therefore, comparing the uptake capacity of megakaryocytes after only 10 days of maturation with full-grown platelets might be an attempt to draw similarities between two things that are not similar.

In conclusion, our data demonstrate that human megakaryocytes cultured in vitro is capable of SERT-selective MIBG uptake. Presumably, the most mature megakaryocytes in the bone marrow are primarily responsible for this uptake. The concomitant application of a SSRI in neuroblastoma patients treated with [^131^I]MIBG therapy seems a promising strategy to prevent radiotoxic MIBG uptake by these cells and the onset of thrombocytopenia. Future studies should examine the effect of citalopram on platelet count, reconstitution, and need for transfusions in [^131^I]MIBG-treated neuroblastoma patients in a placebo-controlled clinical trial.

## Supplementary Information

**Additional file 1**. Panel A. Flow cytometry dot plots of three megakaryocytic cell culture batches (MK1-3) showing expression of the cell surface antigens CD42a, glycoprotein IX (GP9), a marker of true megakaryocytic commitment, and CD42b, glycoprotein Ib, a marker of more mature megakaryocytes. Results were gated for the CD34-negative and CD41a-positive cell population. MK3 has both the most megakaryocytes (57.1%) and the most mature megakaryocytes (22.8%) after 9 days of differentiation. Panel B. Flow cytometry analysis of megakaryocytic differentiation and maturation showing the expression of the major cell surface antigens used to characterize various stages of megakaryocytic differentiation (CD34, CD41a, CD42a, and CD42b). Note: Donor-dependent variation exists in the megakaryocytic differentiation and maturation process. Data from multiple donors was used to create this figure. Panel C. Phase-contrast microscopy of a differentiated megakaryocyte forming proplatelets after 10 days of differentiation. The proplatelets are visible as multiple platelet-sized beads connected together by thin cytoplasmic bridges.

## Data Availability

The datasets generated during and/or analyzed during the current study are available from the corresponding author on reasonable request.
