# Supplementary material for: Thrombocytopenia after meta-iodobenzylguanidine (MIBG) therapy in neuroblastoma patients may be caused by selective MIBG uptake via the serotonin transporter located on megakaryocytes
Source: EJNMMI Res. 2021 Aug 23;11:81. doi: 10.1186/s13550-021-00823-5 (PMC8382772; doi:10.1186/s13550-021-00823-5)

Supplementary figure 1. Panel A: Flow cytometry dot plots of three megakaryocytic cell culture batches (MK1-3) showing expression of the cell surface antigens CD42a, glycoprotein IX (GP9), a marker of true megakaryocytic commitment, and CD42b, glycoprotein Ib, a marker of more mature megakaryocytes. Results were gated for the CD34-negative and CD41a-positive cell population. MK3 has both the most megakaryocytes (57.1%) and the most mature megakaryocytes (22.8%) after 9 days of differentiation. **Panel B**: Flow cytometry analysis of megakaryocytic differentiation and maturation showing the expression of the major cell surface antigens used to characterize various stages of megakaryocytic differentiation (CD34, CD41a, CD42a, and CD42b). Note: Donor-dependent variation exists in the megakaryocytic differentiation and maturation process. Data from multiple donors was used to create this figure. **Panel C**: phase-contrast microscopy of a differentiated megakaryocyte forming proplatelets after 10 days of differentiation. The proplatelets are visible as multiple platelet-sized beads connected together by thin cytoplasmic bridges.

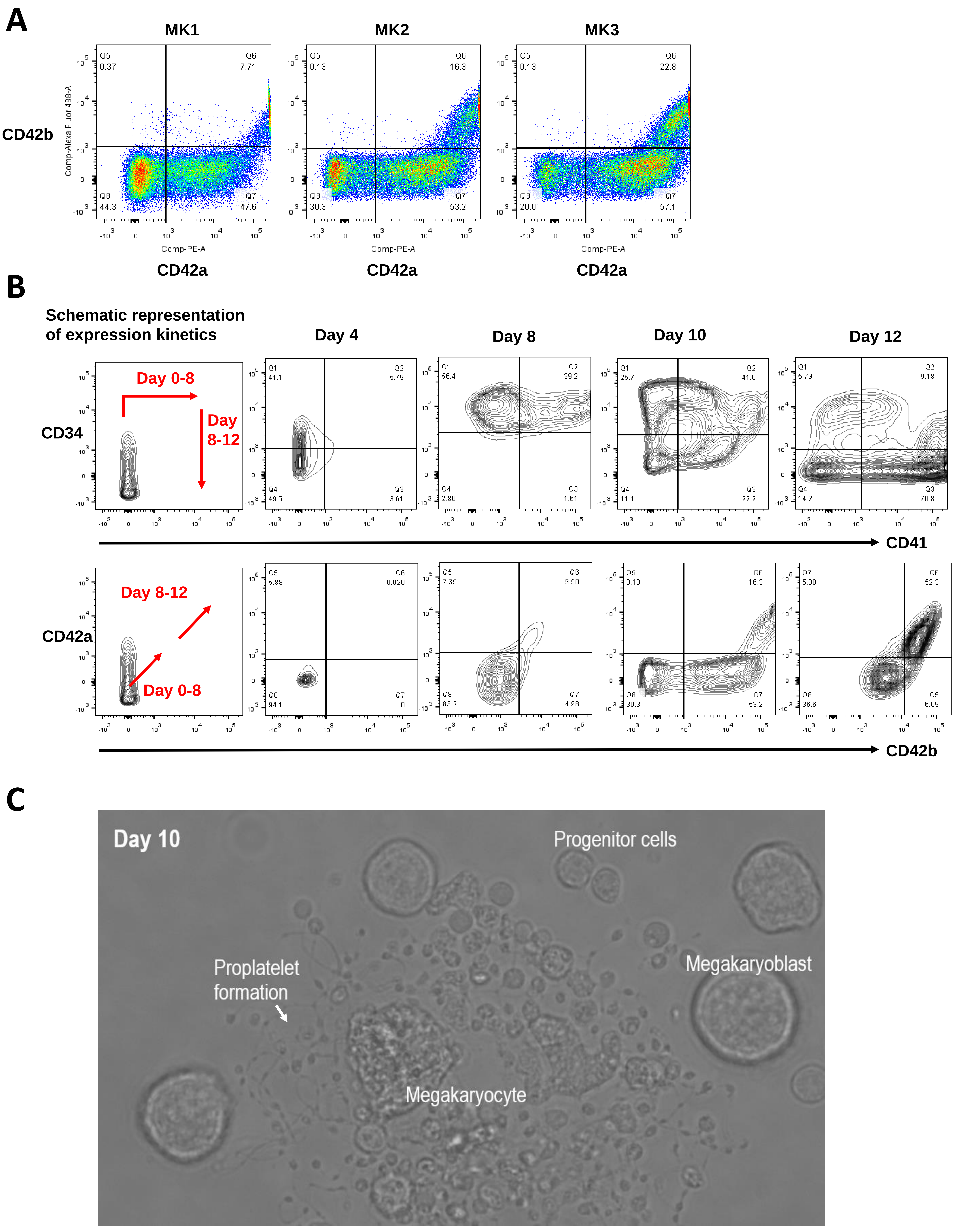

Supplement: Supplementary file 1 — Additional file 1. Panel A. Flow cytometry dot plots of three megakaryocytic cell culture batches (MK1-3) showing expression of the cell surface antigens CD42a, glycoprotein IX (GP9), a marker of true megakaryocytic commitment, and CD42b, glycoprotein Ib, a marker of more mature megakaryocytes. Results were gated for the CD34-negative and CD41a-positive cell population. MK3 has both the most megakaryocytes (57.1%) and the most mature megakaryocytes (22.8%) after 9 days of differentiation. Panel B. Flow cytometry analysis of megakaryocytic differentiation and maturation showing the expression of the major cell surface antigens used to characterize various stages of megakaryocytic differentiation (CD34, CD41a, CD42a, and CD42b). Note: Donor-dependent variation exists in the megakaryocytic differentiation and maturation process. Data from multiple donors was used to create this figure. Panel C. Phase-contrast microscopy of a differentiated megakaryocyte forming proplatelets after 10 days of differentiation. The proplatelets are visible as multiple platelet-sized beads connected together by thin cytoplasmic bridges. [file 13550_2021_823_MOESM1_ESM.pdf]
